# Cortical signatures of visual body representation develop in human infancy

**DOI:** 10.1038/s41598-023-41604-5

**Published:** 2023-09-07

**Authors:** Jiale Yang, Natasa Ganea, So Kanazawa, Masami K. Yamaguchi, Joydeep Bhattacharya, Andrew J. Bremner

**Affiliations:** 1 School of Psychology, Chukyo University, Nagoya, Japan; 2 Child Study Center, Yale University, New Haven, CT USA; 3 Department of Psychology, Japan Women’s University, Tokyo, Japan; 4 Department of Psychology, Chuo University, Tokyo, Japan; 5 Department of Psychology, Goldsmiths, University of London, London, UK; 6 Centre for Developmental Science, School of Psychology, University of Birmingham, Birmingham, UK

**Keywords:** Human behaviour, Perception

## Abstract

Human infants cannot report their experiences, limiting what we can learn about their bodily awareness. However, visual cortical responses to the body, linked to visual awareness and selective attention in adults, can be easily measured in infants and provide a promising marker of bodily awareness in early life. We presented 4- and 8-month-old infants with a flickering (7.5 Hz) video of a hand being stroked and recorded steady-state visual evoked potentials (SSVEPs). In half of the trials, the infants also received tactile stroking synchronously with visual stroking. The 8-month-old, but not the 4-month-old infants, showed a significant enhancement of SSVEP responses when they received tactile stimulation concurrent with the visually observed stroking. Follow-up experiments showed that this enhancement did not occur when the visual hand was presented in an incompatible posture with the infant’s own body or when the visual stimulus was a body-irrelevant video. Our findings provide a novel insight into the development of bodily self-awareness in the first year of life.

## Introduction

As an adult, perceiving one’s own body is essential for skilled interactions with the external world and plays a fundamental role in the sense of self (e.g.^1,2^), and depends extensively on integrating information across multiple senses^3,4^. Studies in recent years have revealed that several brain areas and networks are specialised to respond to bodily multisensory information and encode bodily self-awareness (see, e.g.^5,6^). However, little is known so far about the development of the neural basis of body representations and bodily awareness in early human development (although see^7,8^). In this study, we attempt to establish a neural marker of bodily awareness that can be used to trace the developmental origins of the sense of body ownership and self-awareness in preverbal human infants. Building on research showing that tactile stimulation of the hand promotes visual awareness of one’s body in adults^9^, we investigated putative measures to track the development of bodily awareness in the first year by comparing tactile modulations of steady-state visual evoked potentials (SSVEPs) in 4- and 8-month-old infants.

Researchers interested in the development of body representations in the human brain have recently explored the origins of somatotopy in cortical responses to tactile stimuli^7,10,11^. Studies using a variety of imaging modalities demonstrate that even preterm foetuses have spatial differentiation of cortical hand and foot touch responses to that observed in older infants and adults^10,12^. However, although somatotopy is likely as fundamental to bodily perception as it is pervasive throughout the mammalian nervous system^13,14^, we might have reasonable doubts as to whether such a structure is sufficient to support the rich and dynamic representations of the body required for self-awareness and skilled action (see, e.g.^1,2^). Certainly, the multisensory basis of bodily perception is absent in such somatotopic markers. Studies in adults have demonstrated that perception of one’s body is highly dependent on integrating bodily information across multiple senses (for reviews, see^3,4^).

Several behavioural studies have probed the postnatal development of the multisensory links involved in body representations (e.g., visual-proprioceptive and visual-tactile links; for reviews, see^15,16^). It is now generally established that even with only a few months (or even days) of postnatal experience, human infants are sensitive to the spatiotemporal congruency between cues about the body coming from different senses, specifically between proprioception and vision^17–21^, and between touch and vision/audition^22–27^. Additionally, these types of multisensory links are not limited to one’s own body but can extend to another’s body. For example, at 4 months of age, the somatosensory cortex can be activated by seeing other people being touched^28^.

However, despite indications of an early developing capacity to perceive the multisensory basis of the body, it remains unclear whether these multisensory abilities are related to self-awareness or a representation of one’s own body. For example, an important study on the neural basis of body representations in early infancy^24^ has reported the activation of multisensory association areas in a single age group of 5-month-old infants in response to tactile-visual synchrony. However, whether this multisensory activity is specifically related to body representations is unknown. We attempt to address this gap by conducting three independent experiments.

Given the role of primary visual cortices in multisensory integration^29–31^ and subjective perceptual experience^32,33^, visual cortical responses provide a promising marker for developing self-awareness and body representations. Indeed, somatosensory influences on the visual system can play a crucial role in the calibration of visual estimates of the size and shape of objects^34–38^. For example, a study in adults has shown that when tactile strokes applied to a hand are congruent with strokes observed via visual input concerning an artificial hand, this increases the dominance of the visible hand percept in continuous flash suppression^9^.

In the present study, we investigated the tactile modulation of visual cortical responses to bodily stimuli in a cross-sectional comparison of 4- and 8-month-old infants. The design we used was similar to Van der Hoort et al.^9^ in that we measured whether infants showed greater sensitivity to visual stimuli depicting a body part (a hand) when congruent tactile stimulation was also presented. However, rather than examining visual sensitivity through a verbally reported task, we investigated whether tactile stimulation would modulate the amplitude of SSVEPs in response to the visual presentation of a hand. SSVEPs are a neural correlate of visual awareness in adults^39^, and have been successfully applied in infants^38,39^ and even in newborns^40^. We conducted three experiments in which the visual displays of the body varied in the degree to which they were perceptually compatible with the infant’s own body across multiple senses.

In Experiment 1, infants watched a video of a hand stroked with a metal tube flashing at 7.5 Hz to elicit 7.5 Hz SSVEPs to be measured over the visual cortex. While observing the video, the infants either received a concurrent stroke on their hands (the tactile-visual condition) or did not (the baseline condition). Because synchronous tactile-visual stimulation increases visual awareness of the artificial hand in adults^9^, we proposed that a stronger SSVEP response in the tactile-visual condition than in the baseline condition would indicate tactile facilitation of awareness of visual bodily stimuli. To test whether this SSVEP enhancement is driven by a multisensory body representation that underpins the perception or by merely detecting the tactile-visual contingency of the stimuli, we conducted two further experiments in which we presented a body-irrelevant visual stimulus (Experiment 2) or an inverted visual hand (Experiment 3).

Recent studies show that newborns can detect aspects of multisensory congruence that specify their own body^41^. However, several studies doubt that human infants are bestowed with a fully-fledged sense of their own bodies before postnatal experience^8,42,43^. For example, it has been shown that the referral of tactile stimuli on the hand to coordinates in an external visual space emerges between 4 and 6 months^43^. Additionally, the use of body representations in the service of purposeful actions also undergoes significant spatial tuning, which continues well into late infancy^44^. Therefore, we considered two alternative hypotheses about the visual modulation by tactile information. The first hypothesis would suggest the modulation would be seen throughout the first year of life, while the second one indicates this would undergo development between 4 and 8 months of age.

## Experiment 1

**Figure 1:**
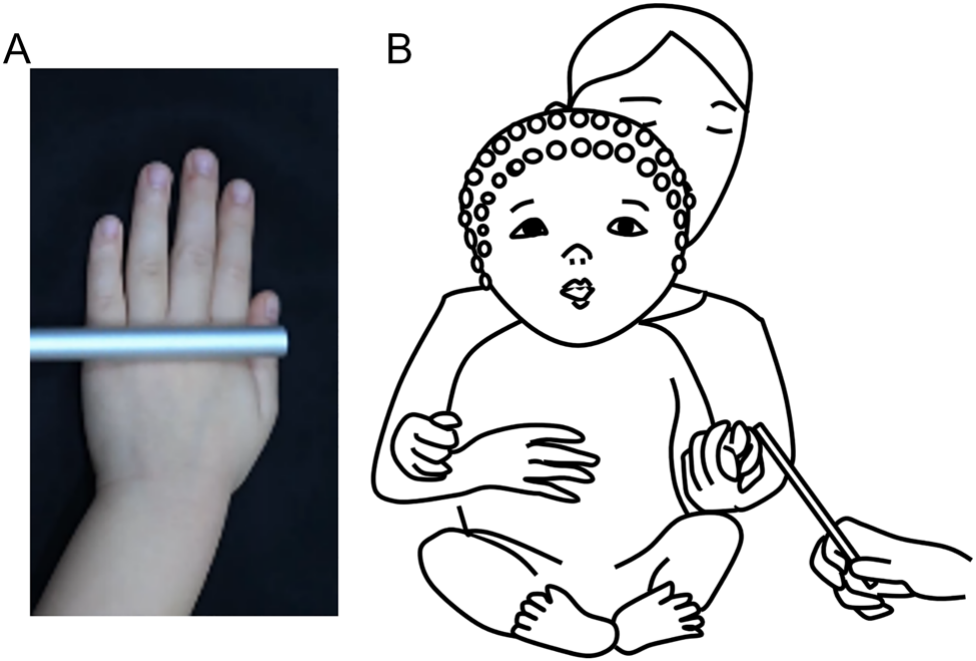
Setup of Experiment 1. (**A**) The visual stimulus was a video in which a left hand was stroked with an aluminium tube. (**B**) tactile-visual condition: Infants watched a flickering (7.5 Hz) video of the hand being stroked accompanied by tactile stroking of their own hand in synchrony with the stroking shown in the video.

We presented groups of 4, and 8-month-old infants with a video of a hand stroked with a metal tube (Fig. 1A) flashing at 7.5 Hz. During viewing this video, infants received an actual concurrent stroke on their hands that was synchronised with the observed strokes made on the screen hand (the tactile-visual condition) (Fig. 1B) or did not receive tactile stimulation (the baseline condition). We recorded EEG simultaneously, expecting to observe SSVEPs at 7.5 Hz over the visual cortex.

### Methods

#### Participants

Fourteen 4-month-old infants (5 male, mean age 127 days; SD = 7.2 days) and fourteen 8-month-old infants participated in this experiment (8 male, mean age 240 days; SD = 10.5 days). We did not perform a power calculation during design because no previous studies were available to estimate the effect size of an enhanced SSVEP response. Therefore, we determined our sample size after previous SSVEP studies with infant participants^45,46^. Ten additional infants were excluded due to fussiness (nine infants) or EEG recording trouble (one infant). All infants were recruited locally, full-term at birth, and healthy at the time of the experiment. The experiment was conducted in accordance with the ethical principles for conducting research with children. This experiment was carried out in the UK, and ethical approval was obtained from the Ethics Committee of the Department of Psychology Goldsmiths, University of London. Written informed consent was obtained from the parents of the participants.

#### Stimuli

Previous studies used various approaches to delivering stimuli to infants, including vibrotactile stimuli^8^, brushes^21–23,41^, mechanical taps^11^, median nerve stimulation^47^, and nociceptive touch^48^. Here, we selected a stimulus that could provide spatiotemporally discriminative cues in both tactile and visual sensory modalities (considering the constraint that the visual ‘on screen’ tactile stimulus was also flickered at 7.5 Hz). We recorded a video in which a toddler’s left hand was stroked with an aluminium tube. The hand was presented on the screen in an upright and dorsal-facing orientation (Fig. 1A). In this video, a slow and gentle stroke was performed on the hand over a 4 cm area, from the wrist to the metacarpophalangeal joint of the middle finger, at a velocity of 4 cm/s moving back and forth across the hand. The stroke was repeated twice, resulting in an overall stimulation duration of 4 s. This video was 320 × 576 pixels and was shown in the centre of the screen at a resolution of 1280 × 960 pixels. At a test distance of 60 cm, the video subtended approximately 8.4 × 15.3 degrees of visual angle. The average luminance per frame of the video was quantified as 38.5 cd/m^2^. This value was derived via the utilization of the standard gamma value and the RGB mixing ratio inherent to the LCD display, along with the maximum luminance values provided by the device manufacturer. The video was presented on a black background and flickered at a frequency of 7.5 Hz. The on/off cycle was 50/50, i.e., a pattern onset/offset mode. In the tactile-visual condition, infants watched the video accompanied by an actual stroke to their left hands synchronised with the video. These strokes were synchronous and spatially congruent with the moving tube shown in the video display. This synchronous presentation was delivered by a trained experimenter, who listened to a click track that contained an auditory cue regarding the direction and velocity of each stroke through headphones. However, if the infants looked away from the monitor, it was possible that they could observe the tactile strokes applied to their hands. Therefore, we excluded those trials from the analyses in which they orientated their fixation to their hands. In the baseline condition, there was no tactile stimulation.

#### Apparatus and procedure

Each infant sat on their parent’s lap throughout the experiment in a dimly lit and sound-attenuated room. Parents were instructed not to interact with their babies. A 24-inch LCD display (width × height: 530 mm × 300 mm, 1920 × 1080 pixels, refresh rate = 60 Hz) was used to present visual stimuli. The infant’s looking behaviour was monitored by a hidden infrared camera set below the display. A dynamic cartoon was shown in the centre of the monitor before each trial to attract the infant’s attention. A second experimenter outside the room observed the infant’s behaviour via a video monitor and controlled the timing of the stimulus presentation after confirming that the infant was looking at the cartoon. The tactical-visual and baseline conditions were randomly presented. The duration of each trial was fixed at 4 s. During the inter-trial intervals, looming images of animals were presented to attract the infant’s attention. Continuous EEG was recorded throughout. Data collection continued as long as the infants remained settled and were attentive to the visual stimulus.

#### EEG recording and analysis

The electrical activity of the brain was continuously recorded using a Hydrocel Geodesic Sensor Net, consisting of 128 silver–silver chloride electrodes evenly distributed throughout the scalp. The electrical potential was amplified by Net Amps 300 (Electrical Geodesics, Inc., Eugene, USA), digitised at a 500 Hz sampling rate, and stored on a computer disk for offline analysis.

A technical colleague who did not know the identity of the stimulus coded the looking behavior of each infant and removed the epochs in which the infant did not fixate the visual stimulus on the monitor for 3.5 s continuously in a single test. This criterion ensured that the EEG signals used for further analysis were recorded when the infants actively observed the visual stimulus. Infants were only included in the further analysis if they completed five or more trials for each condition, a similar criterion used in previous infant SSVEP studies^46^.

The EEG signals were preprocessed using the EEGLAB toolbox in Matlab R2018a (MathWorks, USA). A 1–100 Hz bandpass filter (basic linear finite impulse response filter) was applied to remove slow drifts and high-frequency noise in the EEG signals. The EEG signals were then re-referenced to an average reference and segmented into epochs that lasted from 0 to 4 s following the onset of the visual stimulus. Epochs containing large artefacts (± 250 μV, 3% of trials) were discarded. The segmented data were then averaged in the time domain, separately for each condition and each infant. A discrete Fourier transformation analysis was performed for the averaged data on the electrodes in an EEG frequency domain at a frequency resolution of 0.25 Hz (= 1/4 s). Signal-to-noise ratios (SNRs) were computed for noise variations across the EEG spectrum. SNRs in the 4 s epochs were calculated as the 7.5 Hz signal amplitude ratio and the average of the amplitudes in 8 surrounding frequency bins. The surrounding frequency bins were separated by units according to frequency resolution (0.25 Hz) and comprised windows of ± 1 Hz around the following points: 6.5 Hz, 6.75 Hz, 7 Hz, 7.25 Hz, 7.75 Hz, 8 Hz, 8.25 Hz, and 8.5 Hz)^49,50^.

### Results and discussion

For each condition, we had between 5 and 16 valid trials for analysis (on average, 10.6 trials of tactile-visual condition and 11.4 trials of baseline condition in 4-month-old infants; 8.1 trials of tactile-visual condition and 9.9 trials of baseline condition in 8-month-old infants). The average numbers of trials excluded because the infants looked away from the visual stimulus in the tactile-visual condition were 10.5 (4-month-old infants) and 9.6 (8-month-old infants). For baseline trials, an average of 11.4 (4-month-old infants) and 9.4 (4-month-old infants) trials were excluded for the same reason. There was no significant difference in the number of valid trials contributed in the two conditions between age groups (tactile-visual condition, *χ*^2^ (1) = 0.000, *p* = 0.995, *n.s.*; baseline condition, *χ*^2^ (1) = 0.000, *p* = 1.000, *n.s.*)*.*

**Figure 2:**
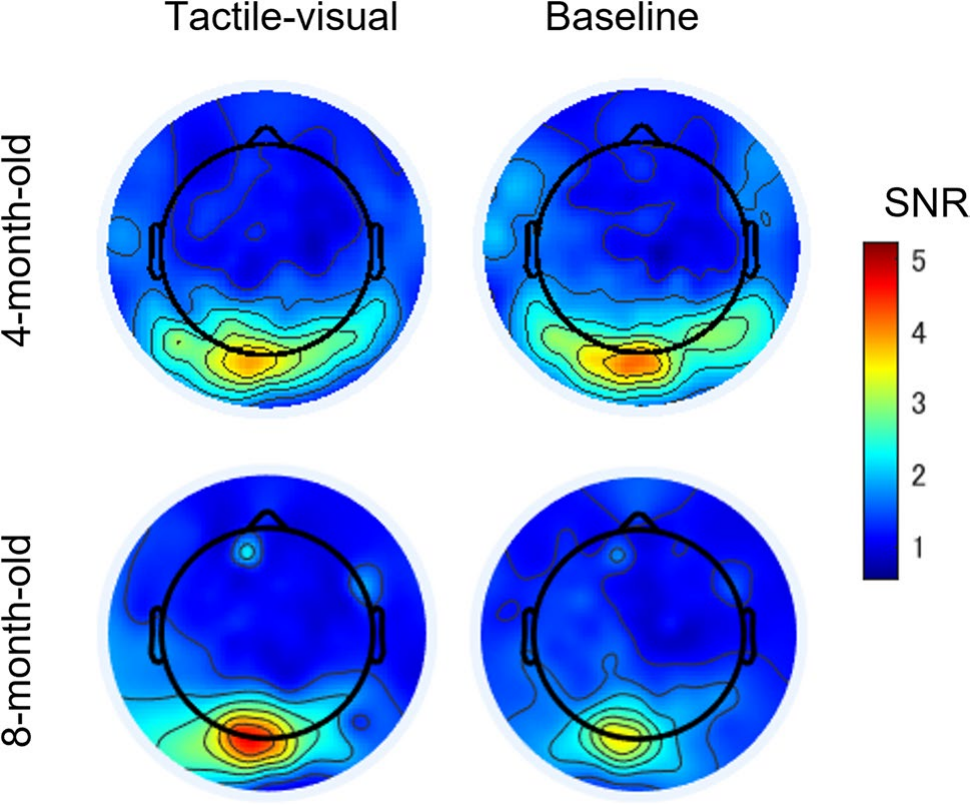
Topographic maps of signal-to-noise ratio (SNR) at 7.5 Hz across age groups (4- and 8-month-old infants) and conditions (tactile-visual and baseline).

**Figure 3:**
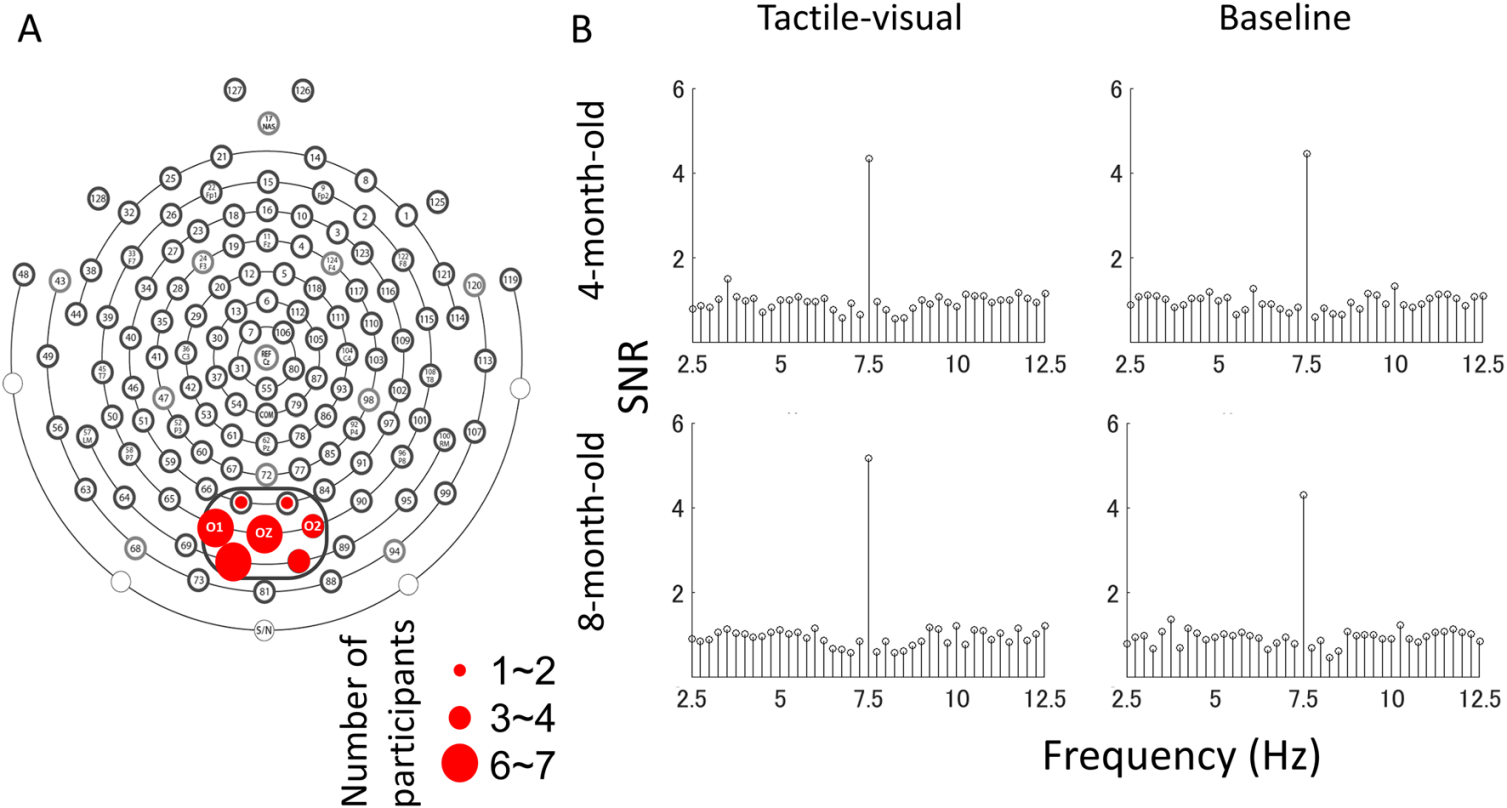
The layout of the Hydrocel Geodesic Sensor Net and the averaged EEG signal-to-noise ratio (SNR) spectrum. (**A**) A circle surrounds the electrodes that were selected for analysis. These electrodes are located in the medial occipital area and cover O1, Oz, and O2 in the international electrode placement system of 10–20. The champion electrodes with the highest SNR at 7.5 Hz were selected for further analysis. The size of each red circle represents the number of participants who showed the highest SNR in the corresponding channel. (**B**) The SNR spectra are shown in age groups and conditions. The SNR spectrum was computed as the ratio of the amplitude at each frequency bin and the 8 surrounding frequency bins at the champion electrodes.

We expected to find strong SSVEP responses in the occipital area because the early visual cortex is a major source of SSVEPs^51^. Therefore, we examined the scalp distribution of the SSVEP response in two conditions (Fig. 2). When we examined the SNR topography at 7.5 Hz, we observed strong SSVEPs in the medial occipital cortex in 4 months and 8 months of infants, indicating that responses in the visual cortex were synchronised with the stimulus flicker rate in both age groups. To determine which electrode exhibited the most synchronisation with the flickered video in each participant, we averaged the raw EEG signal of two conditions for each participant and computed the SNRs for seven electrodes (electrodes 70, 71, 74, 75, 76, 82, and 83 according to the Hydrocel Geodesic Net system) located in the medial occipital area, which covered O1, Oz, and O2 in the 10–20 international system (Fig. 3A). Of note, the same electrodes were also used for the successive experiments in this study. The champion electrodes, which had the highest 7.5 Hz SNR in each participant, were then taken to represent the SSVEP response and used for further analysis. The numbers of participants that showed the highest SNR in the corresponding channel are plotted in Fig. 3A.

In the next analysis, we averaged the raw EEG signal of the champion electrodes and computed the SNR spectrum under each condition for each participant. Figure 3B shows the averaged spectrum from 2.5 to 12.5 Hz in 0.25-Hz steps in the tactile-visual and baseline conditions. To ensure that these electrodes exhibited a significant EEG signal synchronised with the flickering frequency, we compared the individual SNR values at 7.5 Hz against 1 (i.e., the noise level) using one-sample *t*-tests. This analysis revealed that all SNRs were reliably higher than the noise level (for 4-month-old infants: tactile-visual, *t*(13) = 7.027, *p* = 0.000, *d* = 1.878; baseline, *t*(13) = 5.074, *p* = 0.000, *d* = 1.356, and for 8-month-old infants: tactile-visual, *t*(13) = 8.930, *p* = 0.000, *d* = 2.387; baseline, *t*(13) = 8.363, *p* = 0.000, *d* = 2.235; a Bonferroni corrected significance level of 0.0125 was used) (Fig. 3, panels B). These results indicate that the neural response in the visual cortex synchronised strongly with a 7.5 Hz flickering video of a hand stroked.

**Figure 4:**
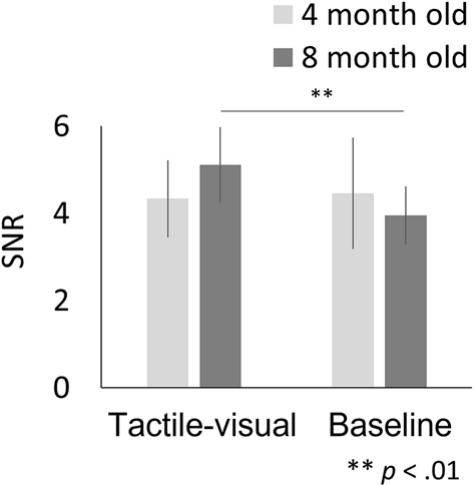
Mean SNR at 7.5 Hz under all conditions (tactile-visual/baseline). The error bars indicate the standard error of the mean. The results for 4-month-old infants are indicated by light gray bars, and those for 8-month-old infants are indicated by dark grey bars. Asterisks indicate statistically significant differences: **p < 0.01.

The final stage of our analysis was to examine the effect of the experimental manipulation on the SSVEP response. SNRs were analysed using a 2 × 2 mixed analysis of variance (ANOVA) with condition (tactile-visual vs. baseline) and age group (4-month-olds vs. 8-month-olds) as factors. This analysis revealed a significant interaction between the factors, condition x age group (*F*(1,26) = 5.223, *p* = 0.031, *η*_*p*_^2^ = 0.167), but no significant main effect of condition or age group (condition, F (1,26) = 3.331, *p* = 0.079, *η*_*p*_^2^ = 0.114; Age group, *F*(1,26) = 0.042, *p* = 0.840, *η*_*p*_^2^ = 0.002)*.* To scrutinize the interaction effect, separate one-way ANOVAs examined the effect of the condition for each age group. For 8-month-old infants, the mean SNR obtained in the tactile-visual condition (M = 5.115, SD = 1.724) was significantly higher (*F*(1, 13) = 15.46, *p* = 0.001, *η*_*p*_^2^ = 0.543) than that in the baseline condition (M = 3.953, SD = 1.321) (Fig. 4). On the contrary, for 4-month-old infants, the mean SNR obtained in the tactile-visual condition, M = 4.334, SD = 1.775, did not differ statistically from that obtained in the baseline condition, M = 4.464, SD = 2.554 (*F*(1,13) = 0.073, *p* = 0.791, *η*_*p*_^2^ = 0.006)*.* We also report Bayes Factor (BF) in order to help evaluate the null hypothesis. A BF exceeding 3 signifies substantial evidence favouring the alternative hypothesis (H1) over the null (H0), often analogous to *p* < 0.05. A BF of 1/3 or less indicates substantial evidence for H0 over H1, while a BF ranging between 3 and 1/3 suggests the data is inconclusive for distinguishing either hypothesis. Following the execution of a Bayesian paired T-test, our findings provide moderate evidence supporting the absence of SSVEP enhancement in 4-month-old infants (BF = 0.279). Conversely, among 8-month-old infants, strong evidence endorses the presence of SSVEP enhancement (BF = 24.7).

Thus, although we could observe SSVEPs in response to a flickering image of a hand being stroked in 4- and 8-month-old infants, only the 8-month-old group showed an improvement in their SSVEPs when tactile stroking was presented simultaneously to their own hand. Next, we examined the specificity of this crossmodal enhancement of visual processing by touch in two additional experiments in which we varied the degree to which the visual stimulus was congruent with the infant’s own body.

## Experiment 2

In Experiment 2, we attempted to replicate the crossmodal influence of touch on visual processing observed in Experiment 1 in a new group of 8-month-old infants. However, instead of presenting a flickering video of a hand being stroked, we presented a body-irrelevant video of a doll dancing on a ball, flickering again at 7.5 Hz. If the tactile enhancement of visual processing observed in 8-month-olds in Experiment 1 was not specific to body representations, we would expect to replicate the tactile enhancement of visual processing.

### Materials and methods

#### Participants

Twelve 8-month-old infants participated in the study (7 males, mean age 245 days; SD = 5.6 days). Four additional infants were excluded from the sample due to fussiness (three infants) and difficulty recording EEG (one infant). All infants were recruited from the UK and were full-term at birth and healthy at the time of the experiment. Ethical approval was obtained from the Ethics Committee of the Department of Psychology at Goldsmiths University of London. In addition, written informed consent was obtained from the parents of the participants.

#### Stimuli

The visual stimulus was a body-irrelevant video in which a doll danced on a ball. The average luminance per frame of the video was quantified as 64.0 cd/m^2^. The video had an identical visual angle to that used in Exp. 1 and flickered at the same frequency, i.e., 7.5 Hz. In the tactile-visual condition, the infants watched the video accompanied by a tactile stimulation identical to that in Exp. 1. In contrast, no tactile stimulation was presented in the baseline condition.

#### Apparatus and procedure

This experiment was carried out in the same laboratory as in Experiment 1. EEG recording and analyses were the same as described in Experiment 1.

### Results and discussion

**Figure 5:**
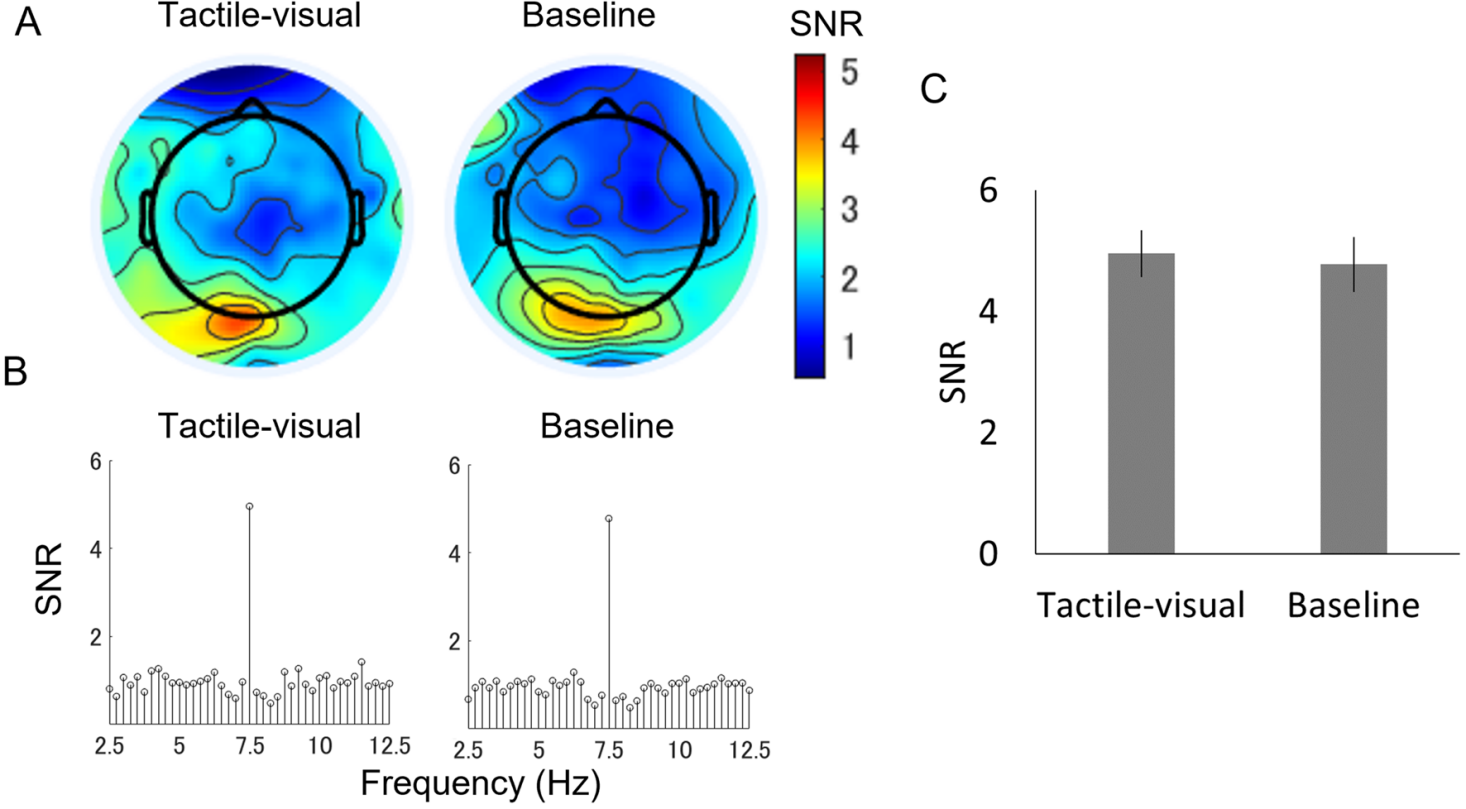
Results of Experiment 2. (**A**) Topography maps for signal-to-noise ratio (SNR) at 7.5 Hz. (**B**) The SNR spectrums under two conditions (tactile-visual/baseline). (**C**) Mean SNR at 7.5 Hz under all conditions. The error bars indicate the standard error of the mean.

The 8-month-old infants participated in between 6 and 20 trials in each condition (on average, 12.3 trials of tactile-visual condition and 14.0 trials of baseline condition; there were no significant condition-wise differences in the number of valid trials contributed, *χ*^2^ (1) = 0.000, p = 1.000, *ns*)*.* The average number of trials excluded because infants looked away from the visual stimulus was 7.3 in the tactile-visual condition and 7.0 in the baseline condition. Consistent with Experiment 1, strong SSVEP responses were observed over the medial occipital cortex, as shown in the SNR topography (Fig. 5A). The champion electrodes also showed a high SNR against noise level (visual-tactile, *t*(11) = 6.784, *p* = 0.000, d = 1.958; baseline, *t*(11) = 9.989, *p* = 0.000, *d* = 2.884, a Bonferroni corrected significance level of 0.0125 was used) (Fig. 5B). A repeated-measure one-way ANOVA with a factor of experimental condition was applied to SNR values to examine the effect of the condition (tactile-visual vs. baseline). No significant effect of the experimental condition was found in Experiment 2 (*F*(1,11) = 0.117, *p* = 0.739, *η*_*p*_^2^ = 0.010). Additionally, a BF of 0.302 from the Bayesian paired T-test provided support the null hypothesis (H0). Consequently, the mean SNR of the tactile-visual condition, M = 4.957, SD = 2.020, did not differ statistically from that of the baseline condition, M = 4.775, SD = 1.309 (Fig. 5C).

It is noteworthy that the SSVEP response in the baseline condition in Experiment 2 (4.775) was larger than that in Experiment 1 (3.953). This increase in response can be attributed to the higher luminance of the visual stimuli in Experiment 2 (64.0 cd/m^2^), compared to Experiment 1 (38.5 cd/m^2^). Given that the visual stimuli were modulated in a pattern of onset/offset, the magnitude of SSVEP response primarily depends on the luminance change between the onset/offset alternation. Consequently, we refrained inferential statistical comparisons of the SSVEP responses in Experiment 1 and 2 within an omnibus analysis.

To summarize, unlike Experiment 1, no enhancement of visual processing was observed under tactile condition, indicating that tactile enhancement of visual processing in infants aged 8 months, which we observed in Experiment 1, could be specific to visual processing of body-related information.

## Experiment 3

In this experiment, we further probed the extent to which the tactile enhancement of visual processing observed in 8-month-olds in Experiment 1 was specific to the visual processing of their own bodies. The design of Experiment 3 was identical to Experiment 1, except the visual stimulus of the hand was inverted, which means that its visually presented posture was incompatible with the posture of the infant observer’s own body. Such manipulations are known to undermine a sense of body ownership in adults (e.g.^52^), and sensitivity to crossmodal visual-tactile congruency in both infants and adults^27,41,53,54^. Therefore, we predicted that if the tactile enhancement of visual processing observed in 8-month-olds in Experiment 1 was driven by a multisensory representation of their own body (rather than representations of bodies more generally), we would see a weaker or absent influence of tactile stimulation on infant SSVEPs.

### Materials and methods

#### Participants

Fourteen 8-month-old infants participated in the study (7 males, mean age 241 days; SD = 11.6 days). Four additional infants were excluded from the sample due to fussiness (three infants) and poor EEG signals (one infant). All infants were full-term at birth and healthy at the time of the experiment. This experiment was carried out in Japan, and ethical approval was obtained from the Ethics Committee of Chuo University.

#### Stimuli

Visual and tactile stimuli were identical to those used in Exp. 1, except that the video stimuli were inverted so that the hand was presented with a finger pointing downward in a posture incompatible with the posture of the infant’s own body.

#### Apparatus and procedure

A 32-inch LCD display (1920 × 1080 pixels, refresh rate = 120 Hz) was used to present visual stimuli. The sizes of the visual stimuli were rescaled so that these subtended a visual angle with the visual stimuli in Experiments 1 and 2. We used different Hydrocel geodesic sensor nets to record the EEG signals. These nets were newer than those used in Experiments 1 and 2. Otherwise, the procedures were the same as those used in Experiments 1 and 2. As before, the tactile-visual and the baseline conditions were presented randomly. The EEG recording and analyses were the same as described in Experiment 1 and 2.

### Results and discussion

**Figure 6:**
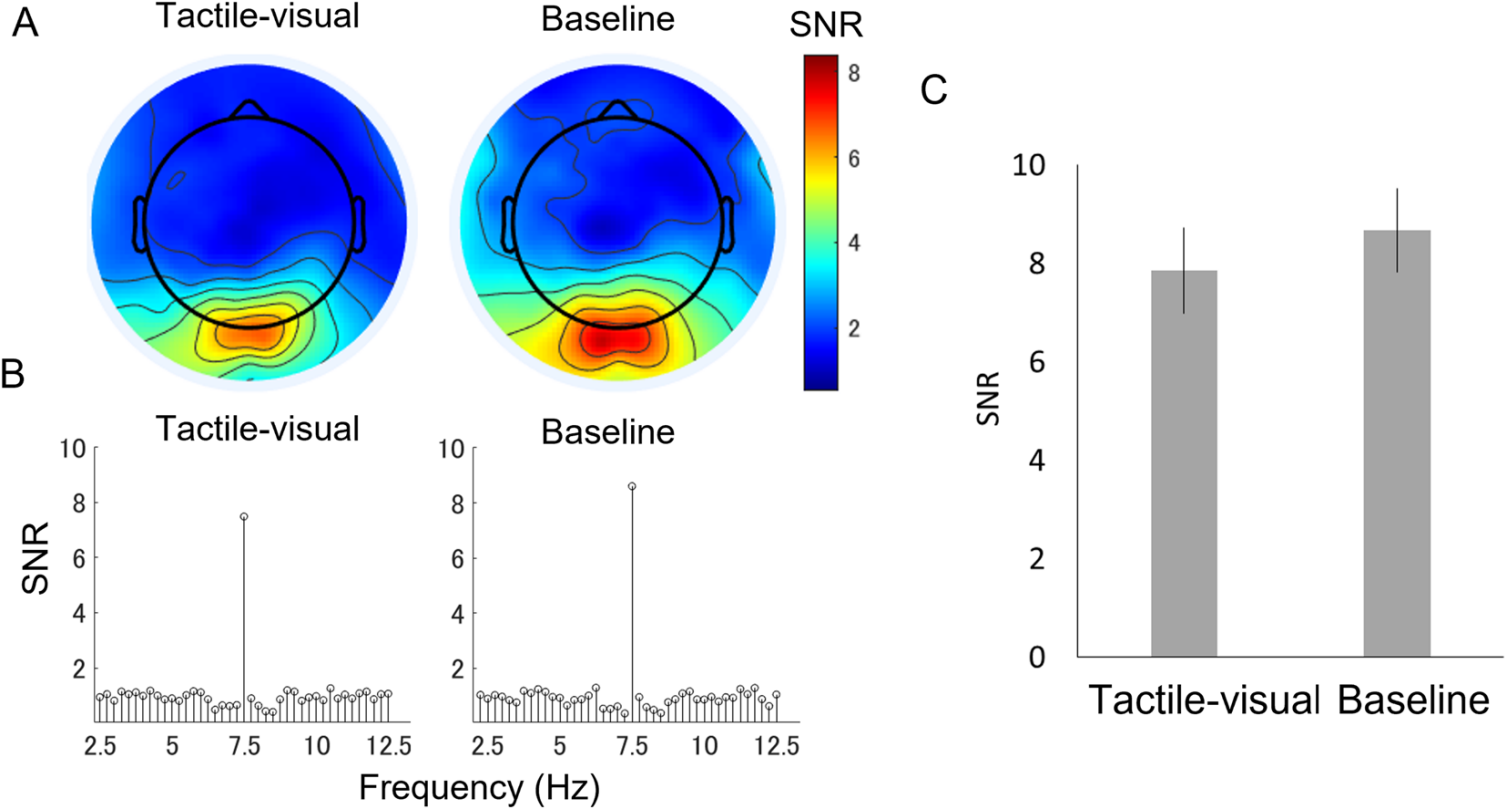
Results of Experiment 2. (**A**) Topography maps for signal-to-noise ratio (SNR) at 7.5 Hz. (**B**) The SNR spectrums under two conditions (tactile-visual/baseline). (**C**) Mean SNR at 7.5 Hz under all conditions. Error bars indicate the standard error of the mean.

The 8-month-old infants contributed between 5 and 22 trials in each condition (on average, 10.9 trials of tactile-visual condition and 12.9 trials of baseline condition; there was no significant difference in the number of valid trials contributed, *χ*^2^ (1) = 0.004, p = 0. 951, *ns*)*.* The average number of trials excluded because infants looked away from the visual stimulus was 10.8 in the tactile-visual condition and 10.3 in the baseline condition. Consistent with Experiments 1 and 2, strong SSVEP responses were observed from the SNR topography in the medial occipital cortex (Fig. 6A). The champion electrodes also had a high SNR against noise level (visual-tactile, *t*(13) = 7.831, *p* = 0.000, *d* = 2.093; baseline, *t*(13) = 9.065, *p* = 0.000, *d* = 2.423, a Bonferroni corrected significance level of 0.0125 was used) (Fig. 6B). New Hydrocel geodesic sensor nets were uesd in Experiment 3. We believe this explains why higher SNR of EEG signals were recorded in Experiment 3 compared to Experiments 1 and 2. A mixed two-way ANOVA with experiment (Experiment 1 vs. Experiment 3) and condition (tactile-visual vs. baseline) was performed to compare the SSVEP values between Experiments 1 and 3. ANOVA revealed a significant interaction between experiments and conditions (*F*(1, 26) = 7.212, *p* = 0.012, *η*_*p*_^2^ = 0.217) and a significant main effect of the experiment (*F*(1, 26) = 17.96, *p* = 0.000, *η*_*p*_^2^ = 0.409), but without a main effect of condition (*F*(1, 26) = 0.221, *p* = 0.642, *η*_*p*_^2^ = 0.008). These results indicated that visual manipulations could modulate the tactile enhancement of SSVEPs in two different experiments. A further one-way repeated-measure ANOVA with the condition was applied to examine the tactile enhancement of SSVEPs in Experiment 3. This showed that the effect of the condition was not significant (*F*(1,13) = 1.462, *p* = 0.248, *η*_*p*_^2^ = 0.101), and indeed any trend in this effect was in the direction of greater SNR in the baseline condition, M = 8.672, SD = 3.167, than in the tactilevisual condition, M = 7.856, SD = 3.276 (Fig. 6C). The Bayesian paired T-test rendered a BF of 0.498. While this result does not strongly endorse the null hypothesis, it indicates that H0 is twice as likely as H1.

## General discussion

In this study involving three independent experiments, we used SSVEPs to investigate the development of visual body representation in 4- and 8-month-old infants. In Experiment 1, we found that concurrent tactile-visual stimulation of the hand enhanced SSVEP responses in 8-month-olds but not in 4-month-olds. In Experiments 2 and 3, we established that this enhancement of SSVEPs in 8-month-old infants was only observed when the visual stimulus that caused the SSVEP resembled the infant’s own body (Experiment 2) and was presented in a posture that was spatially compatible with their own body (Experiment 3). Together, these findings demonstrate that tactile-visual crossmodal interactions in the brain become tuned to certain featural and spatial visual cues concerning the infant’s own body between 4 and 8 months of age. Additionally, we believe that this is the first demonstration of crossmodal influences of somatosensory input on visual processing in human infants.

Enhancements in the visual processing of bodily information by tactile input have been observed in adults, but measured through an increased report of visual images of the hand during continuous flash suppression presentations^9^. Such effects have been taken to indicate that spatiotemporally congruent tactile-visual cues of the kind presented here to promote a sense of ownership and visual awareness of the body in adults. However, human infants cannot report their visual awareness. Here we suggest that the tactile enhancements of SSVEPs, as presented here, provide an intuitive and reasonable alternative measure of visual bodily awareness in infants, which can be added to the growing battery of neural measures of perceptual consciousness in infancy^55,56^. In fact, the SSVEP response to visual stimuli is correlated with visual awareness in adults^39^, and could be enhanced by the visuomotor contingency^57^. We also raise the possibility that the enhancement of SSVEPs could be used as a marker of the sense of limb ownership in infants because visual awareness of the body is correlated with the sense of body ownership in adults^9^. In contrast to 8-month-olds, 4-month-olds in Experiment 1 showed no tactile enhancement of their SSVEPs, leading to the conclusion that the multisensory visual-tactile interactions involved in bodily awareness undergo development throughout the first year of life. Our results undermine the notion that infants are born with fully functional bodily awareness^41,58^, i.e., the notion that the human newborns can be aware of their own body.

In contrast to our findings, two studies have demonstrated neural sensitivity to visual-somatosensory information in early infancy. In a study with 5-month-old infants, Filippetti et al.^24^ reported hemodynamic responses close to the temporal-parietal junction (TPJ) and the superior temporal sulcus (STS) that were sensitive to visual-tactile and visual-proprioceptive multisensory congruency; of note, hemodynamic responses were modulated by the degree of synchrony between the infant’s own bodily movements or tactile experiences and a video feed of their movements viewed on a screen. In a further study with 4-month-old infants, Rigato et al.^28^ reported a modulation of somatosensory evoked potentials according to whether they observed simultaneously the touch, another person’s hand being touched (or the surface next to the hand being touched).

Crucially, neither of these aforementioned studies included a manipulation of the spatial compatibility of the visually observed bodily stimulation with the infant’s own body. Indeed, the argument of Rigato et al.^28^ concerning young infants’ sensitivity to vicariously observed tactile stimulation on another person’s limb is precisely based on the lack of compatibility between the limb of the infant participant and the limb on which they observed the tactile event. As such, although these studies demonstrate infant sensitivity to visual-tactile and visual-proprioceptive congruency in the first 4 to 5 months of age, the conclusions that can be drawn about the presence of representations or self-awareness in these age groups are more limited.

In contrast to the studies just discussed, in our Experiment 1, we saw no evidence that our SSVEP putative marker of visual awareness of the body was modulated by tactile stimulation in our younger sample of infants. This was only seen in the 8-month-old infants’ SSVEP responses when the infants were viewing visual stimuli, which showed the degree of featural and spatial compatibility with the infant’s own body. Therefore, these results suggest the emergence of bodily self-awareness throughout the first postnatal year of life. Further research is required to examine the effects of the specificity of physiological and behavioural measures of self-awareness to multisensory cues specifying the infant’s own body to gain a full picture of the emergence of bodily self-awareness in early life. An intriguing possibility is that infants’ sensitivity to perceptual information about sensory events on other people’s bodies (e.g., as in^28^) may arise earlier than their awareness of their own body^59^.

An important behavioural study in this field has demonstrated an index of visual-tactile perception in newborns modulated by the specificity of visual information concerning the body. Filippetti et al.^41^ found that newborns preferred to look at synchronous visual-tactile stroking on the face (where the newborn viewed another infant’s face on a screen that was stroked in synchrony with their own) compared to asynchronous stroking. Importantly, this effect was not observed when the visual face was inverted, suggesting that attention to visual-tactile congruency was specific to body-specific processing. This effect is comparable to the differential modulation of SSVEPs by tactile stimulation developing between 4 and 8 months in the data reported in Experiments 1 and 3. The crucial difference is that Filippetti’s study involves visual-tactile processing on the face, whereas current studies examine this in relation to the hand. Given that the face is such an important focus of perceptual processing in early postnatal life (e.g.^60^), one potential explanation for the discrepancy in these findings is that bodily tactile-visual processing and awareness may emerge according to different developmental timetables for different parts of the body, with visual-tactile processing of the face forming a primitive and early developing basis for bodily awareness, and awareness spreading to the peripheral limbs as infants gain more competence at controlling these through the first postnatal year. This account would be consistent with an increasing number of studies demonstrating that the development of multisensory limb representation depends on postnatal experience (see, e.g.^8,42,43,61^). For example, several studies now show that the influence of external spatial coordinates on the localisation of tactile stimuli in the hands and feet develops gradually throughout the first year of life^8,42,43,62,63^, and depends on visual experience^61,64–66^.

How does tactile-visual stimulation promote visual processing of the hand in 8-month-old infants? Evidence from recent developmental studies has revealed that multisensory experiences obtained from their own bodies facilitate the development of body representations in infants (for a review, see^16^). We propose that multisensory integrative mechanisms that construct a body representation, rather than tactile stimulation itself, modulate the activity observed in the visual cortex. This view is in line with evidence from human adults showing that activations in the visual cortex, including the primary visual cortex^67^ and the extrastriate body area (EBA)^68–70^, are upregulated by tactile stimulation during a form of rubber hand illusion. In these studies, feedback signals from the intraparietal sulcus and the premotor cortex, which are known to be involved in the sense of body ownership^70,71^, are integrated with a body representation that improves the processing of visual stimulation through a top-down modulation that can play a role in improving visual awareness of the stimulation. This proposal is also compatible with the findings that interregional functional connectivities in the brain develop dramatically after six months of age, while intraregional functional connectivities emerge at birth^72^. Furthermore, recent studies have shown that feedback processing in the visual system develops after 7 months of age^73,74^. Therefore, it is possible that the 4-month-old infants in Experiment 1 did not show any enhancement in SSVEPs due to a lack of feedback from higher-level cortices to the visual cortex, which developed between 4 and 8 months of age.

It is important to carefully qualify the extent to which the data presented here demonstrate clear evidence of the development of bodily self-awareness. In the absence of direct measures of conscious awareness in human infants (i.e., in the absence of verbal report), our approach in this study adopts a natural kinds framework (see, e.g.^75^) in which we seek to identify measures which can comprise a network of measurable phenomena which can lead to more firm conclusions about the origins of awareness of the bodily self in infancy. We have identified SSVEP responses that are measurable in infants as a proxy for visual awareness^33^. Based on this hypothesis, we have examined whether dimensions of bodily stimulation (visual-tactil synchrony, visual orientation of the hand related to the body) known to be predictive of body ownership and visual self-awareness in adults^9^ would also influence the strength of SSVEPs in infants. Given the modulations of SSVEPs between age groups and experiments related to these bodily dimensions, we conclude here that tactile-visual crossmodal interactions in the brain tuned to sensory information specifying the infants’ own bodies develop between 4 and 8 months of age. Importantly, there are further questions that can be asked about the specificity of tactile enhancement of SSVEPs in human infants (such as whether they are specific to other visual cues specifying the infant’s own body such as spatial proximity to the infant, and whether visual-tactile temporal synchrony is required). However, the manipulations undertaken in our report go beyond previous studies of the neural basis of early body representations (e.g.^24,28^), in that they show that the tactile-visual interactions observed are specific to an upright visual orientation of the hand which through its postural compatibility with the infant’s own body preferentially specifies the infant’s own body.

In summary, our findings indicate that multisensory tactile-visual interactions in SSVEPs tuned to sensory information specifying an infant’s own body develop between 4 and 8 months of age. We propose that tactile enhancement of SSVEPs specific to the infant’s own hand in a posturally-compatible upright visual presentation represents a plausible marker of emerging awareness of the bodily self in early life, which can contribute to the growing literature on the origins of conscious awareness in preverbal postnatal development and independently provide a promising avenue for investigating the specificity of early body representations in human infancy.

## Data Availability

The data sets from the current study are freely available from the corresponding author upon reasonable request.
