# Author Correction: Cortical signatures of visual body representation develop in human infancy

**DOI:** 10.1038/s41598-023-46310-w

**Published:** 2023-11-06

**Authors:** Jiale Yang, Natasa Ganea, So Kanazawa, Masami K. Yamaguchi, Joydeep Bhattacharya, Andrew J. Bremner

**Affiliations:** 1 School of Psychology, Chukyo University, Nagoya, Japan; 2 Child Study Center, Yale University, New Haven, CT USA; 3 Department of Psychology, Japan Women’s University, Tokyo, Japan; 4 Department of Psychology, Chuo University, Tokyo, Japan; 5 Department of Psychology, Goldsmiths, University of London, London, UK; 6 Centre for Developmental Science, School of Psychology, University of Birmingham, Birmingham, UK

Correction to: *Scientific Reports* 10.1038/s41598-023-41604-5, published online 07 September 2023

Natasa Ganea was omitted from the author list in the original version of this Article.

The Author Contributions section now reads:

“J.Y. and A.J.B. developed the study concept. All authors contributed to the study design. The testing and data collection were performed by J.Y. and N.G. J.Y. performed the data analysis and interpretation under the supervision of J.B. and A.J.B. J.Y. drafted the manuscript, and N.G., S.K., M.K.Y., J.B., and A.J.B. provided critical revisions. All authors approved the final version of the manuscript for submission.”

The original Article has been corrected.

